# Prediction of the Satisfaction With the Student Life, Based on Teaching Competence and Satisfaction With the School

**DOI:** 10.3389/fpsyg.2019.02506

**Published:** 2019-11-21

**Authors:** Raúl Baños, Antonio Baena-Extremera, María del Mar Ortiz-Camacho

**Affiliations:** ^1^Department of Physical Education and Sports Science, Autonomous University of Baja California, Ensenada, Mexico; ^2^Department of Didactic of Corporal Expression, Faculty of Education Sciences, University of Granada, Granada, Spain

**Keywords:** teacher competences, classroom satisfaction, life satisfaction, student, physical education

## Abstract

The aim of this article was to assess how students evaluate the professional personality competence of physical education teachers in high school and its relation to student satisfaction and student satisfaction with life itself. In line with these aims, this study was completed as a cross-sectional study, which was carried out in a group of 890 physical education students. Of the study group, 50.3% were female and 49.7% were male. The average age was 15.49 years for females (SD 1.79) and 15.00 years for males (SD 2.00). We used a questionnaire featuring the *Physical Education Teacher Competence*, *Intrinsic Satisfaction Classroom Scale*, and *Satisfaction with Life Scale*. The results are presented as descriptive statistics, correlations, and a structural equation modeling analysis showing students’ perceived competence, predicted self-determined satisfaction, which in turn corresponds to life satisfaction.

## Introduction

In recent years, diverse research has highlighted the importance of studying the life satisfaction of students, for the academic and personal repercussions that these carry (Méndez-Giménez et al., 2017; Salvador-Ferrer, 2017). For that, several authors (Diener, 1984; Diener and Emmons, 1985; Argyle, 1987) have used the theory of subjective well-being to study the satisfaction of people, identifying two dimensions namely: cognitive and affective dimensions. With this theory, subjective well-being can be understood as a theoretical construct that contemplates the combination of the cognitive process (judgments of satisfaction and dissatisfaction) and two affective processes (positive affect and negative affect) (Diener and Emmons, 1985). On one side, the affective dimension can be identified with emotions and affections, such as worry or boredom among others. On the contrary, the cognitive dimension is related to evaluative judgments of life satisfaction and its specific areas (Diener and Emmons, 1985). In line with this, Campbell et al. (1976) related the cognitive and affective dimensions of the theoretical construct, with life satisfaction and happiness, respectively. A decade later, Diener et al. (2003) synthesized that subjective well-being can be identified with components of life satisfaction, with satisfaction in specific areas of life and with the affect thereof (positive and negative).

On the other side, existing literature shows that school is an important factor determining happiness in teenagers, since they invest most part of their life in the academic environment (Roeser and Eccles, 2000). The relationship between life satisfaction and the diverse variables of school and educations, such as academic commitment (Lewis et al., 2011), perceived academic competence (Suldo and Huebner, 2006), auto efficiency (Maddux, 2002), disruptive behavior (Suldo and Huebner, 2004), and the academic performance (Gilman and Huebner, 2006; Vecchio et al., 2007), have been extensively researched, finding contradictory results regarding this variable.

It has been proven that the satisfaction/dissatisfaction with physical education (PE) predicts a positive or negative satisfaction with school (Baena-Extremera and Granero-Gallegos, 2015) and that music students obtain higher values of life satisfaction than those who study mathematics, geography, or language (Gültekin and Aricioğlu, 2016). This therefore opens up a new field of study, due to the existence of a relationship between satisfaction on several subjects, with the academic environment and even academic performance (Baños et al., 2019).

To understand this relationship, understanding the motivational theories is fundamental. Among these theories is the self-determination theory (Deci and Ryan, 1985), along with the variables that underline it. For example, Granero-Gallegos et al. (2012) found that students with high levels of satisfaction/or those that view PE as a fun subject were highly intrinsically motivated individuals, who valued the effort and work required in PE, with the objective to improve and to give more value to the subject. In line with this, Baños et al. (2017) show that when a student experiences a class situation where fun and/or boredom is promoted, it has a direct influence on the student’s performance and can have successful results on their learning process or on the contrary lead to the abandonment of the education system. In this paper, the important role of the professor can be appreciated, as the work design can be a key element in the abandonment or the academic commitment of students (Catano and Harvey, 2011). With this in mind, the influence of the teacher is hugely relevant, as the teacher can have a direct influence on school satisfaction (Baena-Extremera and Granero-Gallegos, 2015) as well as the life satisfaction of a student.

In relation to this, the quality of a students’ educational experience is greatly influenced by the teacher’s experience inside the classroom (Hill et al., 2003; Yair, 2008; Baena-Extermera et al., 2013) and their teaching skills. However, it is well known that teaching is multidimensional as well as complex (Wang et al., 2011), where teachers face and respond to a series of factors that could affect the students and the teaching-learning process, for example, the characteristics of the course, classroom management, and the teacher-student interaction (Young and Shaw, 1999; Khine, 2005). Therefore, to improve teacher competence, a series of basic competencies should be broken down, with specific and observable aspects for teachers, that help improve the learning process in the classroom (Denyer et al., 2007) and influences related to aspects of motivation and learning. The efficiency of such competences could vary according to each student’s personality, some requiring a smart teacher, others a perfectionist, or a careful, encouraging, and loving teacher, but above all, a teacher who has the ability to amuse, enthuse, be affective, open, and caring (Moreno, 2009). Competencies like the knowledge a teacher possesses on the subject, the clarity of their presentation, and how they interact with students as well as creativity are positively related to the satisfaction and motivation of students (Sang et al., 2013). Thus, the way in which a teacher plans and organizes PE classes, adapting to the needs of their students, may influence student satisfaction at school (Baena-Extremera and Granero-Gallegos, 2015).

Considering all the above, the importance of this research lies in studying the influence of a PE teacher’s competencies has on student satisfaction. Thus, the hypothesis of this study is that a competent teacher will help students achieve school satisfaction in PE and, at the same time, promote life satisfaction. Considering this hypothesis, the aim of this study is to analyze how a PE teacher’s competencies can predict school satisfaction and, in turn, the life satisfaction of secondary school students.

## Methods

### Participants

The selection of the sample was of a non-probabilistic type and according to the case on which the students could be accessed. A total of 890 high school (HS) and bachelor (BACH) PE students (442 males = 49.7%, age range = 15.00, DT = 2.00; and 448 females = 50.3%, age range = 15.00; DT = 2.00), belonging to five public and private schools in the Region of Murcia and the Region of Alicante (Spain). In total, 152 students belonged to 1° grade HS (17.1%), 160 (18.0%) of 2° grade HS, 182 (20.4%) of 3° grade HS, 186 (20.9%) of 4° HS, 101 (11.3%) of 1° grade BACH and last, 109 (12.2%) of 2° grade BACH.

### Instruments

The following instruments were used to carry out this investigation.

#### Physical Education Teacher Competence

A validated Spanish version of the *Evaluation of Teaching Competencies Scale* by Catano and Harvey (2011) was used and adapted to PE by Baena-Extremera et al. (2015). The instrument presents eight items that measure the student’s perception of the teacher’s effectiveness. The students were asked to indicate the degree of agreement with the items, and the responses were collected using a scale of polyatomic items ranging from low (1-2), medium (3-4-5), and high (6-7). The eight constructs that this instrument evaluates are communication, conscience of work, creativity, feedback, individual consideration of the student, professionalism, problem resolution, and social conscience.

#### Satisfaction With the School

The questionnaire of intrinsic satisfaction was employed and translated to Spanish and validated by Castillo et al. (2001) from the *Intrinsic Satisfaction Classroom Scale*, by Nicholls et al. (1985), Nicholls (1989), and Duda and Nicholls (1992). This instrument presents eight items that measure the scale of satisfaction with school, with two subscales that measure satisfaction/fun (five items) and boredom with school (three items). The scale was used for the phrase “Tell us your degree of disagreement or agreement in relation with the next affirmations, in reference of all of your school subjects.” Responses were collected through a scale of polyatomic items ranging between 1 (totally disagree) and 5 (totally agree).

#### Satisfaction With Life

The questionnaire used was translated to Spanish and validated by Cabañero et al. (2004) of Diener et al. (1985) and is composed of five items that measure only one factor. This instrument uses a five-point polyatomic scale of items, ranging from (1) totally disagree to (5) totally agree.

### Procedure

The design of this work is non-experimental, sectional, descriptive, and predictive. For the development of this study, written informed consent was obtained from the educational centers, teachers, and parents/tutors, and the intention and objectives of the study was provided.

After obtaining the relevant permissions, data were collected. The participants were informed of the study’s purpose, the voluntary nature of their participation, and the confidential treatment of their answers. They were told that there were no right or wrong answers and were asked for their utmost sincerity.

The questionnaires were completed in a classroom in about 25–30 min with the same researcher always present and who could be consulted regarding any doubts during the process, respecting the declaration of Helsinki (Helsinki Declaration World Medical Association, 2013).

### Data Analysis

An analysis of normality in multiple variants was conducted.

For this, a *normality test based on relative multivariant kurtosis* (RMK) of the PRELIS of the LISREL 9.90 program was conducted. Given the normality, a *Confirmatory Factor Analysis* (CFA) to study the adaptation of such instruments to the samples used in this study was conducted. Multiple reliability and validity indexes were calculated, such as Cronbach’s alpha, the compose reliability, and *average variability extracted* (AVE) for each instrument. A correlation analysis between the instruments used was carried out afterwards, as well as diverse models of structural equations, to answer the objective of this study. The calculations were carried out with the statistical package SPSS v.11 and LISREL 8.80.

## Results

### Analysis of Data Normality

In Table 1, we can observe the normality data of the measuring instruments, where finally, the data prove abnormal behavior. The values of RMK for the Evaluation of Teaching Competencies Scale ranged from 1.488 and from 1.123 to 1.237 for the satisfaction with school and life satisfaction, respectively.

**Table 1:** Values of the multivariate normality test.

	Multivariant normalized Kurtosis	Mardia-based-Kappa	Higher limit	Lower limit
ECTS	51.2439	0.488	1.027	0.973
SWS	6.5740	0.147	1.034	0.966
SWL	14.788	0.237	1.045	0.955

*ECTS, Evaluation of Teaching Competencies Scale; SWS, satisfaction with school; SWL, satisfaction with life*.

### Confirmatory Factor Analysis

First, the CFA of each instrument to determine the validity and reliability of the sample used in this study was conducted. The results (Table 2) were acceptable within the limits established in *x*^2^/gl (Bentler, 1989; Tabachnick and Fidell, 2007), in GFI (Hooper et al., 2008), CFI, IFI, NFI, NNFI (Hu and Bentler, 1995), and RMSEA (Cole and Maxwell, 2003; Chen et al., 2008).

**Table 2:** Adjustment fit indices of each model.

	*x*^2^	gl	*x*^2^/gl	*p*	GFI	CFI	IFI	NFI	NNFI	RMSEA
ECTS	60.45	27		0.000	0.99	0.98	0.98	0.96	0.97	0.03
SWS	78.78	19	4.14	0.000	0.99	0.94	0.94	0.92	0.91	0.068
SWL	18.42	5		0.002	0.99	0.99	0.99	0.98	0.98	0.05

*x^2^, Chi-square; gl, degrees of freedom; p, significance; GFI, goodness-of-fit index; CFI, comparative fit index; IFI, adjustment index; NFI, normalized adjustment index; NNFI, non-normative adjustment index; RMSEA, root-mean squared approximation; ECTS, Evaluation of Teaching Competencies Scale; SWS, satisfaction with school; SWL, satisfaction with life*.

### Reliability and Validity Analysis

As some dimensions of the instruments are composed by only a few items, the Cronbach’s alpha represents some limitations according to Ventura-León and Caycho-Rodríguez (2017). For this reason and due to the recommendation of Domínguez-Lara and Merino-Soto (2015), the reliability was calculated by the *ω* of McDonald. Unlike the alpha Cronbach coefficient, it considers the factorial load, which makes the calculus more stable and reflects the true level of reliability without relying on the number of items in the dimension (see Ventura-León and Caycho-Rodríguez, 2017). Values of internal consistency (*ω*) are acceptable between 0.70 and 0.90 (Campo-Arias and Oviedo, 2008), although Katz (2006) can accept values from >0.65.

In Table 3, an analysis of each model is presented: the Cronbach’s alpha values, compound reliability, AVE, the average and typical deviation of Evaluation of Teaching Competencies Scale, satisfaction with school, boredom with school, and life satisfaction. As can be observed, all the indexes of reliability, AVE, and all *α*, which are above the acceptable values according to Dunn et al. (2014) and Hair et al. (2009), emphasize that the compound reliability is considerably more appropriate than the Cronbach’s alpha in ordinal scale types because they do not depend on the number of attributes associated with each concept (Vandenbosch, 1996), as the values are more acceptable for each factor.

**Table 3:** Scale reliability and convergent validity.

	*M*	SD	Composite reliability	AVE	*α*	*ω*
ECTS	5.49	0.96	0.90	0.50	0.85	0.86
SWS	3.16	0.95	0.83	0.50	0.76	0.80
BWS	2.98	0.85	0.75	0.50	0.69	0.71
SWL	3.54	0.87	0.87	0.57	0.83	0.81

*M, mean; SD, standard deviation; AVE, average variance extracted; α, Crombach’s alpha; ω, McDonald’s omega; ECTS, Evaluation of Teaching Competencies Scale; SWS, satisfaction with school; SWL, satisfaction with life*.

### Correlation Analysis

In Table 4, we can see how teacher competence maintains a positive and significant correlation with satisfaction with life (0.119^**^) and satisfaction with school (0.093^**^) but remains negative with the ABU. The satisfaction with school presents a negative and significant correlation with the opposite factor on its scale (−0.546^**^) and positive with satisfaction with life (0.272^**^). The boredom with school finally correlates negative and significantly with satisfaction with life (−0.201^**^).

**Table 4:** Correlation analysis between the variables.

	1	2	3	4
1. ECTS	—	0.093^**^	−0.023	0.119^**^
2. SATD		—	−0.546^**^	0.272^**^
3. ABUE			—	−0.201^**^
4. SATV				—

ECTS, Evaluation of Teaching Competencies Scale; SWS, satisfaction with school; SWL, satisfaction with life. ^*^p < 0.05; ^**^p < 0.01.

### Prediction of Satisfaction With Student Life

**Figure 1:**
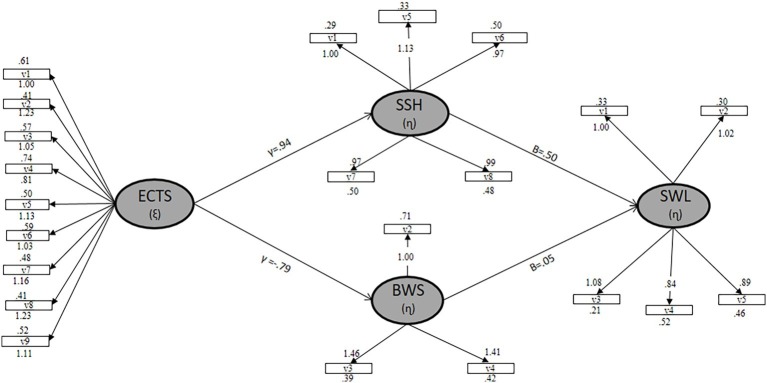
Exogenous variable ECTS (*ξ*), Evaluation of Teaching Competencies Scale; endogenous bariable SSH (*ɳ*), dimension of satisfaction with school; endogenous variable BWS (*ɳ*), dimension boredom with school; endogenous variable SWL (*ɳ*), satisfaction with life; *γ*, gamma values; *β,* beta values.

In accordance with the objective of this study, different models have been hypothesized to test which would adjust better according to the recommendation of Markland (2007). According to the correlations, the model that better predicted the satisfaction with the student life was approved. The structural regression models were evaluated through the combination of the adjustment indexes previously explained, with their values being *x*^2^/gl = 4.32, GFI = 0.96, CFI = 0.92, IFI = 0.92, NFI = 0.95, NNFI = 0.96, and RMSEA = 0.06. These values adjust perfectly to the acceptable parameters (Bentler, 1989; Tabachnick and Fidell, 2007; Hooper et al., 2008, among others). According with the Figure 1, we can observe gamma, beta, lambda-x, lambda-y, and theta delta and theta épsilon values (Figure 1). In it, we can appreciate how the Evaluation of Teaching Competencies Scale positively predicts the impact of the satisfaction with school (*γ* = 0.94), being negative for boredom with school (*γ* = −0.79), satisfaction with school, predicts at the same time the satisfaction with life in a positive way (*β* = 0.50), being the prediction of boredom with school (*β* = 0.05). Finally, the estimated parameters were considered significant when the value associated with value *t* is greater than 1.96 (*p* < 0.05) and all items showed individual reliability values of (*λ*) > 0.05.

## Discussion

The objective of this study was to analyze how a PE teacher’s competencies can predict the satisfaction with school, and how these, at the same time, predict the life satisfaction of HS and BACH students. The importance of this research lies in the influence that a PE teacher can generate on the subjective wellbeing of adolescents, not only in the academic field but also in other areas of life. In addition, adolescence is a stage of life that carries profound changes, in which friend and family influences are transformed and where decision-making has a greater impact on adult life (Maršanić et al., 2014). Failing school and even the increasing suicide rate at this stage of life are fundamental aspects of this study, similar to those already mentioned (Kosik et al., 2017).

We emphasize that the three scales, the Evaluation of Teaching Competencies Scale, satisfaction with school and satisfaction with life, obtained viable and reliable results.

The obtained results in this study demonstrate positive and significant correlations between teacher competencies with satisfaction with school and satisfaction with life and had no correlation with boredom with school, although it did find signs of a limited negative relation. These results showed that a PE teacher who is a good communicator, with work and social conscience, a creative and problem resolution capacity, who provides feedback, who has good individual consideration with a student, and who in conclusion is professional, can affect satisfaction not only through fun and how satisfied they are with school but also can also have repercussions in their satisfaction with life. Similar results were found by Kuzmanovic et al. (2013) who showed that students were satisfied when the teacher was available to solve problems, showing consideration, and providing feedback. So, a good teacher is distinguished by his/her students, as a good stimulator, innovator, enthusiastic, with a good sense of humor, and who is self-reflective, and supportive of diversity (Johnstone, 2005; Khine, 2005; Yair, 2008; Delaney et al., 2009).

Satisfaction with school is significantly positively correlated to satisfaction with life; however, it is significantly negatively correlated to boredom with school, and this, at the same time, is significantly negatively correlated with satisfaction with life. Although it has been proven that teenagers who are perceived as happier, learn faster, behave better, and show greater commitment and academic performance (Noddings, 2003; Ben-Arieh, 2008; Castro, 2011; Baena-Extremera and Granero-Gallegos, 2013; Lyons and Huebner, 2016), the school plays an important role in their subjective well-being, due to the time they spend on academics; therefore, if they find it monotonous and boring, their happiness could be diminished. This input was reflected in the results obtained in this present study, where a student who is unsatisfied at school is related to low levels of satisfaction with life. In line with this, Lyons and Huebner, 2016 found similar results, since students who became bored at school showed less school commitment and more dissatisfaction with their life. Considering this, the correct planning of classroom sessions to avoid monotonous and boring classes is of utmost importance and can increase the levels of satisfaction with life (Quinn and Duckworth, 2007).

In the results of the predictive model, it could be observed how the PE teaching competencies positively predicted the satisfaction with school, and in turn, the satisfaction with life, obtaining similar results to the studies realized by Baena-Extremera et al. (2015). This helped to understand the weight that a PE teacher has on the educative system and on the life of the students, where the difference of being a competent teacher or not, could directly influence the satisfaction or boredom of a student and therefore indirectly influence life satisfaction (Baños et al., 2017). It is also known that the excellence and quality of the institution, as well as the professionalism of the teacher, with traits such as fairness and impartiality, predicts the satisfaction of the student in the classroom and the satisfaction with the teaching quality (Elliot and Shin, 2002) emphasizing the need for good teaching, the setting of clear goals, suitable evaluation methods depending the level of the students, planning of appropriate workload, and having ideal general competencies for teaching (Ginns et al., 2007). However, if teachers do not produce the sufficient impact on their students (Cameron and Lovett, 2015) and the student’s perception of the incompetencies of their PE teacher predicts boredom with school, they will not find the prediction of satisfaction with life. These results are important, as an unsatisfied student increases the possibility of failing school or even abandoning school (Elmore and Huebner, 2010) and increases the probability of suicide in teenagers (Singer et al., 2018). For this reason, it is important to have good teaching practices, emphasizing the influence that a PE teacher can have on their students if they are well prepared, avoiding monotonous classes, with organized planning, where the educator gives autonomy to the students and where sessions approach the students’ interests (Delaney et al., 2009; Calderón et al., 2013; Lee et al., 2015; Lebrero et al., 2019). By doing this, motivation for PE classes will likely improve (Baena-Extremera et al., 2013), affecting the school and faculty (Aubert et al., 2014), improving the results on the PISA report, alarming on the Spanish education system (Casquero and Navarro, 2010).

Finally, the advances presented in this study with relation to previous research can be summarized. A PE teacher can positively predict the life satisfaction of students through the subject they teach and can especially influences stages where they experience big personal changes, morphological and psychological. Therefore, the original contribution of this study is the impact a PE teacher has not only with regard to the physical activity in a teenager’s spare time (Wallhead and Buckworth, 2004; Baena-Extermera et al., 2016) but also on the satisfaction with school and life satisfaction life.

### Limitations and Strengths

Future research should consider using different levels and ages and should even consider analyzing these variables in other subjects. Another limitation of this study is its design; a future possibility is to carry out a quasi-experiment, considering the school organization. Nevertheless, the strength of this study is the large number of participants used and the originality of the investigation as very few studies on this topic are present in the current literature.

## Data Availability Statement

The datasets generated for this study are available on request to the corresponding author.

## Ethics Statement

Ethical review and approval were not required for the study on human participants in accordance with the local legislation and institutional requirements. Written informed consent from the participants was not required to participate in this study in accordance with the national legislation and the institutional requirements.

## Author Contributions

RB and AB-E conceived the hypothesis of this study and analyzed the data. RB, AB-E, and MO-C participated in data collection and wrote the paper with the most significant input from AB-E. All authors contributed to data interpretation of statistical analysis and read and approved the final manuscript.

### Conflict of Interest

The authors declare that the research was conducted in the absence of any commercial or financial relationships that could be construed as a potential conflict of interest.
